# Editorial: Antioxidant properties of phytochemicals

**DOI:** 10.3389/fnut.2024.1354987

**Published:** 2024-03-18

**Authors:** Fujie Yan, Jitbanjong Tangpong, Wei Chen

**Affiliations:** ^1^Department of Food Science and Nutrition, College of Biosystems Engineering and Food Science, Zhejiang University, Hangzhou, China; ^2^Biomedical Sciences, School of Allied Health Sciences, Walailak University, Nakhon Si Thammarat, Thailand

**Keywords:** antioxidant properties, phytochemicals, food chemistry, nutrition, functional food

The articles in this Research Topic entitled “*Antioxidant properties of phytochemicals*” clarify beneficial effects of phytochemicals derived from various food for human health and techniques to increase yield and activity. This research is aimed to feature high-quality advanced research and knowledge suggested by various research groups of the world working on the bioactive properties, bioavailability, physicochemical properties, structural features of phytochemicals. This Research Topic includes three research papers that have undergone multiple rounds of peer review. The main factors affecting acceptance are novelty, research depth, accuracy, and completeness of experimental results, etc.

The first accepted article was submitted by Huang et al. They utilize a series of extraction methods including water extraction with different temperature (room temperature, 80°C, 96–98°C) and ultrasound extraction with water or ethanol (25 and 50%, v/v), to separate and purify polysaccharides from mulberry fruit. In this study, six mulberry fruit polysaccharides (MFPs) were finally obtained which possess different yield, microscopic morphology and monosaccharide composition. MFP-III exhibited the best inhibitory effect on α-glucosidase and α-amylase activity, whereas MFP-IV showed the strongest antioxidative ability *in vitro*. The results of this study indicated that the chemical structure and biological activities of MFPs were significantly different with diverse extraction methods, which affected their antioxidant and hypoglycemic activities, and provided important references for the application of mulberry polysaccharides in food and pharmaceutical industries.

Advanced extraction technologies for enhancing yield of phytochemicals have been paid more and more attention. Another work in this Research Topic by Wang et al. investigated the extraction process of polyphenols from pomegranate peel. Steam explosion is a typical physicochemical pre-treatment technology that can effectively promote the release of polyphenols and improve the extraction rate, being applied to bioactive ingredients pre-treatments and extraction. For this study, authors compared different parameters including pressure, duration, and sieve fractions in steam explosion on the effect of phenolic release of pomegranate peel. Steam explosion obviously increased the contents of ellagic acid and gallic acid, while decreased the content of punicalin and punicalagin. The best conditions were pressure 1.5 MPa, duration 90 s and 40-mesh for the extraction of total phenols, and 1.5 MPa and 120 s for the extraction of ellagic acid and gallic acid. However, the structural identification of phenolic compounds in pomegranate peels with steam explosion pre-treatment needs to be further explored.

Moreover, the discovery of new phytochemicals is of great significance to the development of functional foods. Zhou et al. identified a large number of metabolites from purple, green, yellow, and white carnation flowers through widely targeted LC-MS/MS-based metabolomics analysis. In comparison with the other colored flowers, 128 key differential metabolites were screened in the purple flowers, being considered to have the highest antioxidant and anticancer activities. 2-Deoxyguanosine was firstly identified to have antiproliferative activity against A549 and U2OS cells and the combination of 2-deoxyguanosine with 6-hydroxykaempferol-3, 6-O-diglucoside, or quercetin-3-O-sophoroside exhibited a better anti-tumor efficacy. The discovery enriches information about edible flower, which will stimulate their consumption and develop innovative ingredients for future flower foods.

In conclusion, many of the phytochemicals from different food sources have good antioxidant activity and are significantly related to the plant variety and extraction method. The exploitation and utilization of these natural food resources is of great significance for promoting human health. Nevertheless, there is still lack of in-depth research on the corresponding relationship between components and functions of bioactive substances in plant-derived food, the extraction methods of natural products need to be further optimized. Studies about bioactive components extraction, analysis and evaluation are necessary, so as to better understand the benefits of phytochemicals.

## Author contributions

FY: Conceptualization, Writing—original draft, Writing—review & editing. JT: Writing—review & editing. WC: Conceptualization, Writing—review & editing.

